# Antibodies Against Human BLyS and APRIL Attenuate EAE Development in Marmoset Monkeys

**DOI:** 10.1007/s11481-012-9384-x

**Published:** 2012-06-30

**Authors:** S. Anwar Jagessar, Nicole Heijmans, Jan Bauer, Erwin L. A. Blezer, Jon D. Laman, Thi-Sau Migone, Matt N. Devalaraja, Bert A. ’t Hart

**Affiliations:** 1 Department Immunobiology, Biomedical Primate Research Centre, PO Box 3306, 2280 GH Rijswijk, The Netherlands; 2 ErasMS Center for Translational research into MS, Rotterdam, The Netherlands; 3 Department of Neuroimmunology, Medical University Vienna, Center for Brain Research, Vienna, Austria; 4 Image Sciences Institute, University Medical Center Utrecht, Utrecht, The Netherlands; 5 Department of Immunology, Erasmus MC, University Medical Center, Rotterdam, The Netherlands; 6 Human Genome Sciences, Rockville, MD USA; 7 Department of Medical Physiology, University Medical Centre Groningen, Oosterparkwijk, The Netherlands

**Keywords:** Non-human primate, Multiple sclerosis, B-cell Depletion, Cytokines, EAE

## Abstract

B lymphocyte stimulator (BLyS, also indicated as BAFF (B-cell activating factor) and CD257), and A Proliferation Inducing Ligand (APRIL, CD256) are two members of the TNF superfamily with a central role in B cell survival. Antibodies against these factors have potential therapeutic relevance in autoimmune inflammatory disorders with a proven pathogenic contribution of B cells, such as multiple sclerosis (MS). In the current study we performed a multi-parameter efficacy comparison of monoclonal antibodies against human anti-BLyS and anti-APRIL in a common marmoset *(Callithrix jacchus)* model of experimental autoimmune encephalomyelitis (EAE). A MS-like disease was induced by immunization with recombinant human myelin/oligodendrocyte glycoprotein (rhMOG) in complete Freund’s adjuvant. The results show that the anti-BLyS and anti-APRIL antibody cause significant depletion of circulating CD20+ B cells, but a small subset of CD20 + CD40^high^ B cells was not depleted. Induction of CD20+ B cell depletion from lymph nodes was only observed in the anti-BLyS treated monkeys. Both antibodies had a significant inhibitory effect on disease development, but all monkeys developed clinically evident EAE. Anti-BLyS treated monkeys were sacrificed with the same clinical signs as saline-treated monkeys, but nevertheless displayed significantly reduced spinal cord demyelination. This effect was not observed in the anti-APRIL treated monkeys. The two antibodies had a different effect on T cell subset activation and the profiles of *ex vivo* released cytokines. In conclusion, treatment with anti-BLyS and anti-APRIL delays the development of neurological disease in a relevant preclinical model of MS. The two mAbs achieve this effect via different mechanisms.

## Introduction

Multiple sclerosis (MS) is an inflammatory demyelinating disease of the human central nervous system (CNS). The disease is pathologically characterized by the presence of demyelinated lesions within the white as well as grey matter, which contain a variable degree of inflammation and damage to axons and neurons (Lassmann et al. 2007). Although demyelinated lesions are likely formed by the synergy of cellular and humoral immune mechanisms, most immunotherapies developed for MS target T cells (Lopez-Diego and Weiner 2008). The results of two recent clinical trials seem to have caused a paradigm shift in therapy development. Unexpectedly, treatment of relapsing/remitting MS (RRMS) patients using an antibody against human anti-IL12p40 aiming at the simultaneous inhibition of Th1 and Th17 T cell responses as demonstrated in psoriasis (Laws and Warren 2011), showed no detectable clinical benefit in RRMS (Segal et al. 2008). On the other hand, treatment of a similar group of patients with monoclonal antibodies against human CD20, causing profound and long lasting depletion of B cells, caused rapid and sustained suppression of neurological symptoms (Barun and Bar-Or 2012).

Total CD20+ B cell ablation is a highly effective manner to induce robust and long-lasting disease suppression in a variety of autoimmune disorders, including rheumatoid arthritis (RA), systemic lupus erythematosus (SLE) (Pers et al. 2008) and MS (Barun and Bar-Or 2012). However, the treatment spares plasma cells it causes impairment of the immune defence against new and latent infections. Occasional reactivation of hepatitis B and C, and polyoma JC virus can give rise to serious clinical complications (Carson et al. 2009). This situation warrants the question whether profound systemic B cell depletion is indeed needed for the therapeutic effect or whether alternative B cell targeting treatments may be equally beneficial.

The primary aim of this study was to test the efficacy of two monoclonal antibodies, one for B Lymphocyte Stimulator (BLyS), also known as B-cell Activating Factor (BAFF, CD257) of the TNF family, and a second for A Proliferation-Inducing Ligand (APRIL, CD256). Both cytokines have an important role in the differentiation of B cells (Daridon et al. 2008). A secondary aim of the study was to obtain mechanistic information on the two antibodies by examining changes of autoimmune reactions. The efficacy of the two antibodies was tested in an experimental autoimmune encephalomyelitis (EAE) model in common marmosets (*Callithrix jacchus*), induced by immunization with recombinant human myelin/oligodendrocyte glycoprotein (rhMOG) formulated in complete Freund’s adjuvant (CFA) (Brok et al. 2000; Kap et al. 2008). This rhMOG/CFA EAE model has been well established as a valid preclinical model of MS for translational research into immunopathogenic mechanisms and the preclinical evaluation of novel immunotherapies (’t Hart et al. 2011).

In a recent set of experiments we used HuMab 7D8, a novel fully human IgG monoclonal antibody (mAb) that binds the same epitope on human CD20 as ofatumumab to examine the pathogenic contribution of B cells to this model (Teeling et al. 2004; Kap et al. 2010). We reported that the profound clinical effect antibody was caused by suppression of cellular and humoral autoimmune mechanisms that lead to lesion formation and neurological deficit (Kap et al. 2010; Kap et al. 2011).

In the here reported study we used essentially the same design for the efficacy testing of the anti-BLyS and anti-APRIL antibodies. Both antibodies induced depletion of CD20+ B-cells, which was reflected by suppressed autoantibody production and modulation of T cell proliferation and cytokine production. Both antibodies induced a significant delay of the disease onset. The clinical effect of the anti-BLyS antibody was stronger than of the anti-APRIL antibody. Treatment with the anti-BLyS antibody also lowered demyelination of the spinal cord, whereas this was not observed in monkeys treated with the anti-APRIL antibody.

## Materials and methods

### Animals

Eighteen adult male common marmosets (*Callithrix jacchus*) were purchased from the outbred colony maintained at the Biomedical Primate Research Centre (Rijswijk, The Netherlands). Individual data of all monkeys used in this study are listed in Table 1. Before inclusion in the study the animals were subjected to a complete physical, haematological and biochemical examination. Only monkeys that were declared healthy by the institute’s veterinarians were used. During the study they remained under veterinary care. Monkeys were pair-housed in spacious cages enriched with branches and padded shelter on the floor. Daily diet consisted of commercial food pellets of New World monkeys (Special Diet Serviced, Witham, Essex, UK), supplemented with raisins, peanuts, marshmallows, biscuits, fresh fruit, maggots and gum. Drinking water was provided ad libitum.

**Table 1:** Overview of marmoset used in this study and the post-mortem MRI read-outs of white (WM) and grey (GM) matter lesions in one cerebral hemisphere

Group	Animal	DOS^a^	Age^b^	MRI lesions					
				Volume (mm^3^)		T_2_ (sec)		MTR (%)	
				WM	GM	WM	GM	WM	GM
Control	M02061	30	102	0	0	–	–	–	–
	M05053	57	62	540.7	34	65.9	71.7	29.3	26.5
	M07052	40	41	0	0	–	–	–	–
	M08071	50	26	0	0	–	–	–	–
	M08103	45	25	0	0	–	–	–	–
	Mi010311	55	114	1.1	0	64.9	–	31.8	–
Mean ± SD		46 ± 10	62 ± 38	270 ± 382	34 ± n/a	65 ± 0.7	72 ± n/a	31 ± 2	26 ± n/a
Anti-BLyS	Mi016	102	153	189.5	10.4	64.2	68	32.3	26.6
	M03163	93	82	0	0	–	–	–	–
	M05025	75	66	330.8	20.0	65.5	70.6	32.4	26.3
	M07088	79	45	0	19.6	–	70.1	–	27.6
	M08099	56	25	0	0	–	–	–	–
	M09017	52	19	398.6	54.7	68.1	75.2	30.7	25.3
Mean ± SD		76 ± 20	65 ± 49	306 ± 107	26 ± 20	66 ± 2	71 ± 3	31 ± 1	27 ± 2
Anti-APRIL	M0169	64	108	170.7	13.4	68.1	73.6	30.4	24.7
	M08029	43	30	186.4	27.9	66.8	76.5	32.0	26.0
	M05049	94	62	28.7	1.1	60.7	72.5	35.0	28.8
	M07036	109	42	1.8	0	–	–	34.1	–
	M08092	52	25	0.4	0	–	–	32.3	–
	M09031	52	18	0	0	–	–	–	–
Mean ± SD		69 ± 26	48 ± 33	78 ± 93	14 ± 13	62 ± 5	74 ± 2	33 ± 2	27 ± 2

All animals were males

^a^Day of sacrifice

^b^Age in months at the start of the experiment

According to the Netherlands’ law on animal experimentation, the procedures of this study was reviewed and approved by the institute’s ethics committee before initiation of the experiments.

### Antigens

Human MOG extracellular domain (rhMOG) was expressed as an unglycosylated recombinant protein in *Escherichia coli* and purified in the BPRC laboratory, as previously described (Kerlero de Rosbo et al. 1997; Smith et al. 2005). All synthetic peptides based on the human MOG sequence, which were used for in vitro assays, were purchased from Cambridge Research Biochemicals Limited (Cleveland, UK).

### EAE induction and clinical scoring

EAE was induced with 100 μg of rhMOG emulsified in CFA as previously described (Kap et al. 2010). All animals were daily monitored for neurological signs using a standard scoring systems (Kap et al. 2008; Jagessar et al. 2010). Briefly, 0 = no clinical signs; 0.5 = apathy, loss of appetite, altered walking pattern without ataxia; 1 = lethargy, anorexia, loss of tail tonus, tremor; 2 = ataxia, optic disease; 2.5 = paraparesis or monoparesis, sensory loss; 3 = paraplegia or hemiplegia; 4 = quadriplegia; 5 = spontaneous death due to EAE. The clinical end-point for each monkey was score 3 and overt neurological symptoms were observed from score 2.

### Experimental design

The study protocol was identical to a prior efficacy evaluation of a fully human anti-CD20 antibody (HuMab7D8) in the same rhMOG/CFA model(Kap et al. 2010). The human anti-BLyS antibody (Benlysta; also known as belimumab) and anti-APRIL antibodies were provided by Human Genome Sciences, Inc. (Rockville, MD). Binding affinities of the anti-BLyS and anti-APRIL mAbs with recombinant human and marmoset BLyS and APRIL were determined by BIAcore analysis. Anti-BLyS bound with similar affinity to human BLyS (Kd 447 ± 30 pM) and marmoset BLyS (Kd 744 ± 32 pM), whereas binding of the anti-human APRIL mAb to marmoset APRIL (Kd 19.7 ± 6.6 nM) was about 8-fold lower than to human APRIL (Kd 2.4 ± 1.4 nM).

All monkeys were randomized to three groups of 6. Anti-BLyS and anti-APRIL mAbs were administered intravenously at a dose of 10 mg/kg (1 ml/kg) once a week from day 21 after immunization until the end of the study. The control group received buffered saline (1 ml/kg) also once per week from day 21 after immunization.

It is important to point out that the genetic heterogeneity of the marmoset implies a highly variable response of individual animals in the model at the clinical, pathological and immunological level. In agreement with guidelines by the institute’s animal experimentation committee we used power calculation to assess the minimal group size for statistical evaluating the treatment on the disease course. An inherent problem of the genetic variation in the model is that underlying immunopathogenic mechanisms are variable and do not develop synchronously. For this reason robust statistical data for secondary disease parameters are often not obtained. This problem that is inherent to preclinical research with higher species has recently been discussed elsewhere (Bacchetti et al. 2011).

### Post-mortem examination

Monkeys selected for necropsy were first deeply sedated by intramuscular injection of alfaxan (10 mg/kg) (Vétoquinol S.A., Magny-Vernois, France). After collection of the maximum venous blood (PBMC) in EDTA vacutainers, animals were euthanized by infusion of sodium pentobarbital (Euthesate®, Aphormo, Duiven, The Netherlands). At necropsy brain and spinal were removed for (immuno)histological examination and magnetic resonance imaging (MRI). Secondary lymphoid organs were aseptically removed for preparation of mononuclear cell (MNC) cultures; axillary (ALN), inguinal (ILN), lumbar (LLN) lymph node and spleen as previously described (Jagessar et al. 2008; Jagessar et al. 2010; Kap et al. 2010; Jagessar et al. 2012b). Femur was collected for isolation of bone marrow (BM) cells. Number of leukocytes, neutrophils and lymphocytes in blood was measured on an automated haematology analyzer (Sysmex XT-2000iV, Norderstedt, Germany).

### T cells proliferation

Proliferation was tested on MNC isolated from venous blood (EDTA) every two weeks or at necropsy from spleen and secondary lymph nodes (ALN, ILN, LLN) against a panel of overlapping MOG peptides (10 μg/ml) and rhMOG (10 μg/ml). Proliferation was expressed as stimulation index (SI), which is defined as the ratio between [^3^H]-thymidine incorporation in the stimulated versus unstimulated cultures (Jagessar et al. 2008). SI values above 2 were considered positive.

### MNC phenotyping

MNC were phenotyped as previously described (Jagessar et al. 2010; Jagessar et al. 2012b) and proliferating cells identified via CFSE vital dye dilution (Kap et al. 2008). Flow cytometry data were collected on a FACS LSRII cytometer and analysed with the FACSDiva software 5.0 (BD Biosciences).

### Cytokine profiling by ELISA

Supernatants of MNC cultures were collected after 48 h stimulation with rhMOG or a panel of overlapping MOG peptides. Supernatants were assayed according to manufacturer’s instruction with commercial available ELISA kits for monkey IFN-γ, monkey TNF-α (U-Cytech, Utrecht, The Netherlands) and human IL-17A (eBioscience, San Diego, CA).

### Quantitative PCR

Total RNA was extracted from PBMC, spleen and ALN using RNAeasy minikit (Qiagen, Hilden, Germany). Subsequently, cDNA was synthesized for qPCR using primer and probe combination according to the Universal Probe Library (Roche, Indianapolis, In) as previously described (Jagessar et al. 2012a). Transcript levels were normalized against the reference gene Abelson (ABL).

### Autoantibody detection

Immune sera were tested for the presence of IgM and IgG antibodies binding rhMOG or a panel of overlapping 23-mer pMOG sequences with ELISA as previously described (Jagessar et al. 2010).

### Statistical analysis

Data are presented as mean ± sem. Statistical analysis was performed using the Mann-Whitney *U* test. Survival was evaluated using Log-Rank test. *p* values ≤ 0.05 were considered significant.

## Results

### Induction of B cell depletion by anti-BLyS and anti-APRIL antibodies

Eighteen unrelated common marmosets were randomized to 3 groups of 6 animals each (see Table 1). EAE was induced in all animals by a single immunization with rhMOG in CFA. Treatment with anti-BLyS and anti-APRIL was started 21 days after the immunization, but before clinically evident EAE was observed. Both antibodies were administered as a weekly intravenous dose of 10 mg/kg (1 ml/kg); the control group received the same volume of buffered saline.

**Fig. 1:**
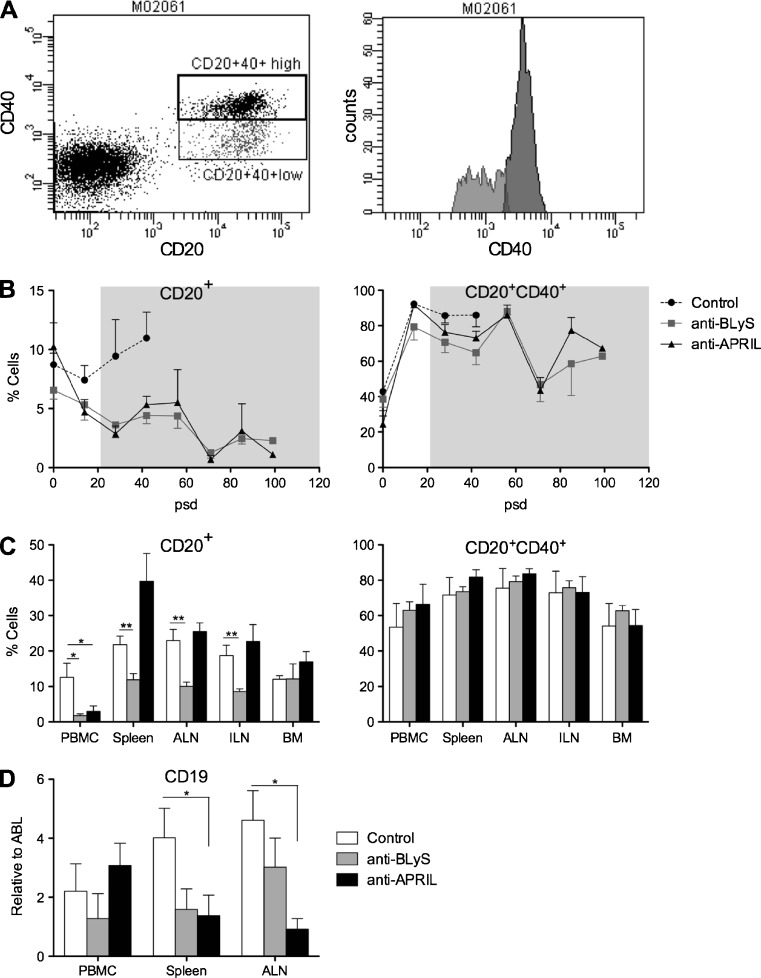
B cell depletion by anti-BLyS and anti-APRIL antibodies. **a.** CD20+ and CD40+ expression by PBMC was analysed by flow cytometry. Based on CD40 expression two populations could be defined, namely CD20 + CD40^high^ (13 %, dark shaded) and CD20 + CD40 + ^low^ (7 %, light shaded). Depicted data are from monkey M02061 as a representative example. **b.** CD20+ (left panel) and CD20 + CD40+ (right panel) expression was measured in PBMC collected at the indicated time points. The CD20 + CD40+ includes the CD20 + CD40 + ^high^ and CD20 + CD40 + ^low^ population The grey shaded area indicates the period of treatment, which was started at post sensitization day (psd) 21. Percentages on the y-axes are cell numbers expressed relative to the total analysed cell number (mean ± SEM). CD20+ B cells are reduced by the anti-BLyS and anti-APRIL treatment compared to untreated monkeys, but this is not the case for CD20 + CD40+ mature B cells. **c.** MNC were prepared from blood, spleen, ALN, ILN and BM at necropsy to measure the presence of CD20+ B cells (left panel) and CD20 + CD40+ mature B cells (right panel). Given percentages on y-axes indicates the fraction of stained cells from the total measured cells (mean ± SEM). In the anti-BLyS treated group a significant decrease of CD20+ B cells was detected in all tested organs except BM. Anti-APRIL treatment had only in blood a significant effect on CD20+ B cells. For the CD20 + CD40+ cells no differences were observed between the control and antibody treated groups. **d.** At necropsy also the expression levels of CD19 mRNA transcripts were measured in PBMC, spleen and ALN, showing a reduction in both antibody-treated group, although the differences with anti-APRIL were only significant for spleen and ALN. Data was normalized to the household gene ABL (mean ± SEM). *p < 0.05 Mann Whitney *U* test, treated group vs. control group

We tested B cell depletion,by flow cytometric analysis of CD20 and CD40 expression on MNC, and qPCR analysis of CD19 mRNA transcripts (Kap et al. 2010). Figure 1a shows subsets of circulating marmoset CD20+ cells expressing low CD40 (±7 %) or high CD40 (± 13 %). Figure 1b depicts the longitudinal analysis of both subsets after immunization with rhMOG/CFA, showing that the percentage CD20+ cells first decreased slightly in control monkeys followed by a clear increase. The percentage CD20+ cells decreased in the groups treated with anti-BLyS and anti-APRIL mAb. Interestingly CD20+ cells expressing high CD40 were not depleted by the treatment.

To assess whether the depletion of B cells was systemic and long lasting we analysed expression of CD20 and CD40 on MNC isolated from blood, spleen, axillary and inguinal lymph nodes and bone marrow collected at necropsy (Fig. 1c). In the anti-BLyS treatment group we observed significant reduction of CD20+ cells in blood and lymphoid organs, but not in the BM. In the anti-APRIL group a significant effect on CD20+ B cells depletion was only observed in blood, whereas the percentage CD20+ cells seemed increased in the spleen. Again, the two antibodies had no detectable effect on the percentage of CD20 + CD40+ cells.

The flow cytometry data for the anti-BLyS treated group were confirmed by the analysis of mRNA transcript levels for CD19 (Fig. 1d). However, we observed an unexplained discrepancy between the increased CD20 staining and reduced CD19 mRNA level in the spleen of anti-APRIL treated monkeys

Taken together these data show that the anti-BLyS antibody consistently decreases B cell number in blood, spleen and lymph nodes, while a variable effect of the anti-APRIL antibody was observed in the monkeys.

### Plasma antibody levels as a surrogate marker of systemic B cell reduction

**Fig. 2:**
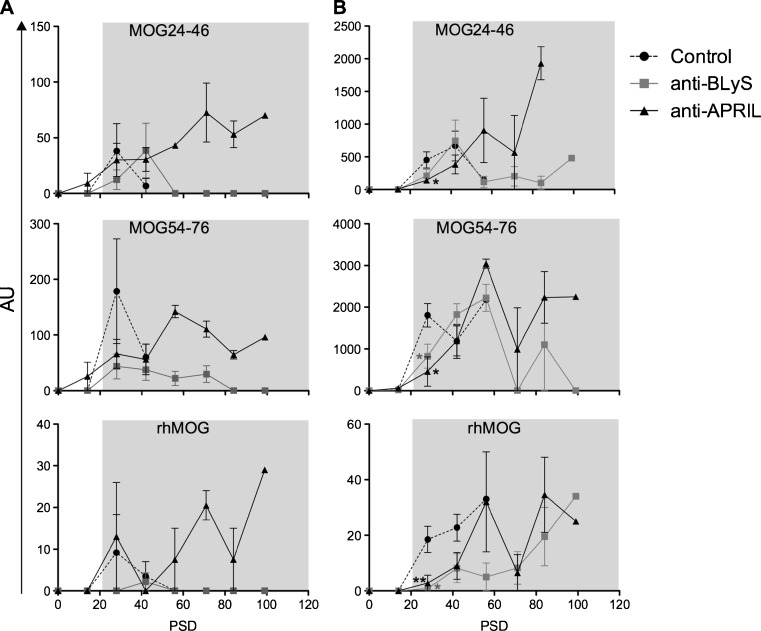
Anti-BLyS treatment reduced antibody production. Plasma samples collected at two weeks interval and necropsy were tested with ELISA for the presence of IgM **(a)** and IgG **(b)** antibodies against the B-cell epitopes MOG24-46 and MOG54-76, and against intact rhMOG. In the anti-BLyS treated group, reduction of IgM and IgG levels were observed during the entire study, but in the anti-APRIL treated groups IgM as well as IgG remained high. Mann Whitney *U* test has been performed but no significantly differences were observed

We tested plasma antibody levels against the immunizing rhMOG protein and two B cell epitope containing peptides, i.e. MOG24-46 and MOG54-76, as an independent biomarker of systemic B cell depletion. Similar to previous studies, IgM serum antibodies against rhMOG in control monkeys peak during a short time interval after the immunization. Serum IgG antibodies come up somewhat later and remain high. Essentially the same patterns are observed for IgM (Fig. 2a) and IgG (Fig. 2b) antibodies against the two peptides. However, the antibody profiles differed clearly between the anti-BLyS and anti-APRIL treated groups. In general IgM antibody levels in the monkeys treated with anti-BLyS mAb remained markedly lower than in the group treated with anti-APRIL mAb. For IgG antibody levels this effect was less clear. The plasma level of anti-rhMOG IgG antibodies was clearly suppressed in the anti-BLyS treated group compared to the control group, whereas an inconsistent variable effect was observed for the anti-APRIL treated group.

### Delayed EAE onset by anti-BLyS and anti-APRIL treatment

**Fig. 3:**
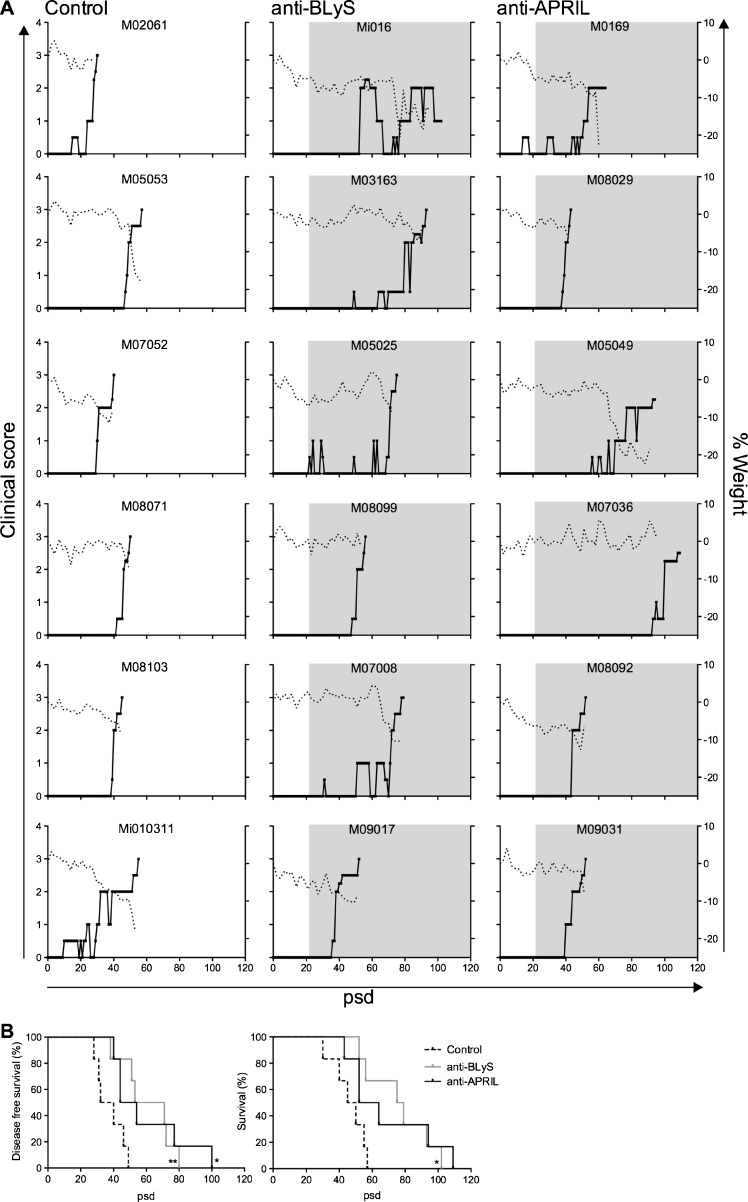
Delay of EAE course by anti-BLyS and anti-APRIL treatment. **a.** Clinical scores are depicted of controls (left panel) and the two antibody treated groups, with anti-BlyS (middle panel) or anti-APRIL (right panel). The solid lines represent clinical scores (left y-axes) and the dotted line body weight loss relative to the immunization day, defined as post sensitization day (psd) 0. Grey shaded boxes indicate the treatment period. All animals in the experiment developed clinically evident EAE (clinical score 2.0) and most of them were sacrificed with an EAE score of 3.0. However, monkey Mi016, M0169 and M05049 had to be sacrificed at an earlier time point due to the serious body weight loss. **b**. Survival curves depict the disease free survival time (time interval to development of EAE score 2.0; left panel) and overall survival (time interval to clinical end point; right panel). Disease free survival times were significantly prolonged in the anti-BLyS and anti-APRIL treated monkeys. The total survival was significantly prolonged by the anti-BLyS treatment, but the delay in anti-APRIL treated monkeys was not significant (*p* = 0,0646). *p < 0.05; **p < 0.001 Log-rank test, treated group vs. control group

All animals in all the three groups developed clinically evident EAE and were sacrificed with a maximum score of 3, which was taken as the predefined ethical end-point. The graphs in Fig. 3a depict the clinical score and body weight loss of individual monkeys relative to the day of EAE induction (psd 0). Some monkeys (Mi010311, Mi016, M03163 and M05049) went into remission after a short period of visual problems reminiscent of optic neuritis. Two animals in the anti-APRIL group (M0169 and M05049) and one in the anti-BLyS group (Mi016) were sacrificed with an EAE score of 2. The reason for sacrifice, before the ethical end-point was reached, was the high bodyweight loss and low physical activity.

Comparison of the time interval to the onset of clinically evident EAE (score 2.0) of the anti-BLyS and anti-APRIL groups with the control group showed a significantly delayed disease onset (disease-free survival curves in Fig. 3b). The subsequent disease progression to the ethical end-point (score 3.0) was also delayed in both groups. However, the delay was statistically significant for the group treated with anti-BLyS mAb, but not for the anti-APRIL treated group (*p* = 0.0646).

### Effect of the anti-BLyS and anti-APRIL mAbs on CNS pathology

**Fig. 4:**
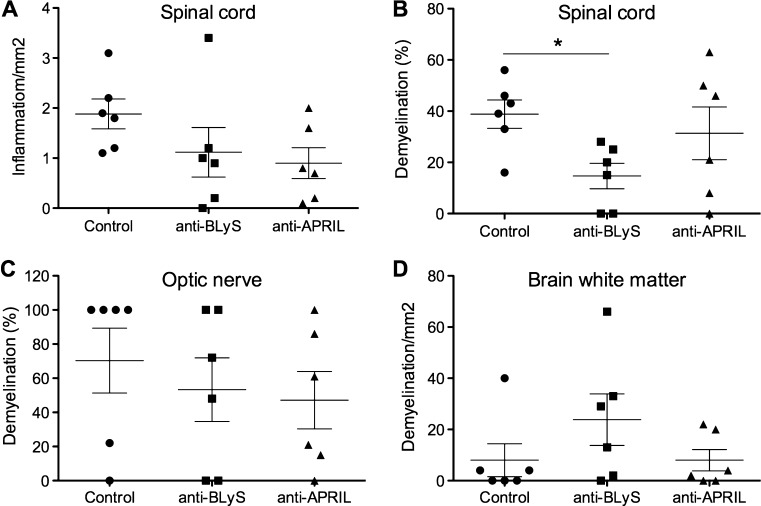
Reduced spinal cord demyelination in anti-BLyS treated monkeys. Formalin-fixed tissue samples were stained to analyse the intensity of inflammation and demyelination. For each animal 8 slices were examined, which equals 6 cm^2^ in total. **a.** The number of infiltrated cells per mm^2^ is given for the spinal cord. The amount of demyelination is given for the spinal cord **(b)**, optic nerve **(c)** and brain white matter **(d)**. A significant treatment effect was only observed for the spinal cord of anti-BLyS treated animals. *p < 0.05 Mann-Whitney U test, antibody treated group vs. control group

Histological analysis of the spinal cord shows reduced inflammation in the anti-BLyS and anti-APRIL group compared to the controls. However, the differences in individual monkeys were too variable to reach statistical significance (Fig. 4a). The histology data also show a higher mean percentage demyelination of the brain in the anti-BLyS treated group, but also these differences were not statistically significant. In contrast, the anti-BLyS treated group displayed a significantly lower severity of spinal cord demyelination, while this was not observed for in the group treated with anti-APRIL mAb (Fig. 4b). Quantification of demyelination of the optic nerve suggested also a protective effect of the anti-BLyS and anti-APRIL mAbs. In the mAb treated groups 2/6 monkeys showed 100 % demyelination of the optic nerve while in the control group there were 4 control monkeys with 100 % demyelination.

In conclusion, the histology data show a remarkable opposite effect of the anti-BLyS treatment on brain and spinal cord demyelination. This was not observed with the anti-APRIL mAb.

### Effect of the mAb on *ex vivo* T cell proliferation

**Fig. 5:**
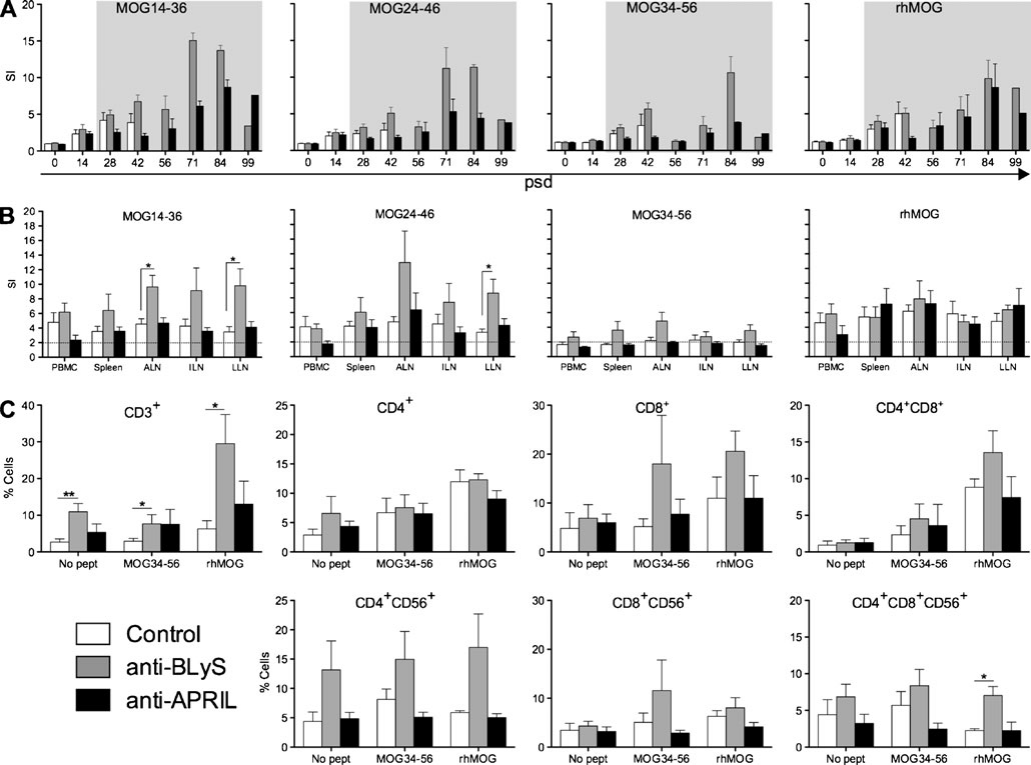
Modulation of T cell proliferation in anti-BlyS and anti-APRIL treated monkeys. **a.** PBMC were isolated at the indicated time points (psd; post sensitization day) and assayed for the response to stimulation with rhMOG or 23-mer synthetic peptides containing previously identified dominant MOG T cell epitopes, i.e. MOG14-36, MOG24-46 and MOG34-56. The read-out was incorporation of [^3^H]-thymidine during the final 18 h of 3 days culture. Data on the left panel show the stimulation index (SI). Grey shaded boxes indicate the period of treatment. T cell proliferation increased after immunization, and was significantly enhanced during the anti-BLyS treatment, but was reduced during anti-APRIL treatment. **b.** At necropsy the incorporation of [^3^H]-thymidine was measured as a read-out for proliferation in PBMC, spleen, ALN, ILN and LLN against the same MOG antigens as described under (A). **c.** Proliferated T cells in blood and lymphoid organs were phenotyped using the CFSE diluting assay. Data are shown of MNC derived from spleen, as a representative example. Stimulation with MOG34-56 and rhMOG are depicted. The y-axes indicates the percentage proliferated cells; CD4+, CD8+ and double positive for CD4 and CD8 were first gated within the CD3+ proliferated population, next for CD4 + CD56+, CD8 + CD56+ and CD4 + CD8 + CD56+, respectively. Data are presented as mean ± SEM. *p < 0.05 Mann Whitney U test, antibody treated group vs. control group

As a read-out for T cell proliferation we determined incorporation of [^3^H]-thymidine (T-cell proliferation assay: TCPA) and CFSE vital dye dilution (Fig. 5).

#### TCPA

Figure 5a shows the longitudinal proliferation of PBMC against the immunizing rhMOG protein, the peptides MOG14-36 and MOG24-46, which contain the MOG24-36 epitope of early acting Caja-DRB*W1201 restricted CD4+ Th1 cells (Brok et al. 2000) and MOG34-56, which contains the MOG40-48 epitope of late acting Caja-E restricted CD4 + CD8+ cytotoxic T lymphocyte (CTL) (Kap et al. 2008; Jagessar et al. 2012b). During the initial 40 days after immunization the differences between the three groups were relatively small. However, at psd 42 we observed a increase of PBMC proliferation in the control and anti-BLyS-treated groups, while proliferation in the anti-APRIL treated group remained low. At 14 days later all control monkeys had been taken out of the experiment with clinically evident EAE. From psd 58 onwards PBMC proliferation in the anti-BLyS group was usually higher than in the monkeys treated with anti-APRIL mAb.

We also determined the proliferative response of MNC from blood and lymphoid organs at the height of the disease (Fig. 5b). The figure shows that the proliferation of MNC from spleen and LLN against the tested MOG peptides is consistently higher than that of the control and anti-APRIL-treated monkeys. No significant difference was found for the MNC proliferation against the immunizing rhMOG protein between the anti-BLyS treated and control monkeys.

#### CFSE dilution

Flow cytometric analysis of CFSE labelled cells for dye dilution in combination with mAb staining was used to assess whether proliferation maps to a certain subset of T cells. We have focused on the response of spleen MNC against MOG34-56 as these contain the T cells that are the most likely culprit for the induction of neurological deficits (Kap et al. 2008) mainly localize in the spleen (Jagessar et al. 2010).

Freshly isolated spleen cells were stained with CFSE and subsequently cultured for 7 days without antigen and with MOG34-56 or rhMOG. Cells harvested from the cultures were incubated with selected monoclonal antibodies and binding was analysed by flow cytometry. The results are given in Fig. 5c. A first remarkable observation was that unstimulated CD3+ spleen MNC from monkeys treated with anti-BLyS mAb contain a significantly higher number of cells with CFSE dilution than CD3+ spleen MNC from control or anti-APRIL treated monkeys. This higher background proliferation in these animals localizes in the CD4 + CD56+ subset. Seven days culture of the spleen cells with MOG34-56 did not detectably enhance proliferation of CD3+ cells and the CD3 + CD4 + (CD56+) subsets in all three groups, but proliferation of CD3 + CD8+ and CD3 + CD8 + CD56+ cells was markedly, albeit non-significantly, enhanced in the anti-BLyS treated group. By contrast, 7 days stimulation of spleen cells with rhMOG induced significantly enhanced proliferation of CD3+ spleen cells, which localized to the CD3 + CD8+, CD3 + CD4 + CD8+ and CD3 + CD4 + CD8 + CD56+ subsets.

Collectively, these data show that the treatment with anti-APRIL had a less outspoken modulatory effect on the analysed cellular responses than the anti-BLyS treatment. This is in accordance with the postulate that the primary focus of APRIL activity is in the humoral arm of the immune system, whereas BLyS is involved in cellular as well as humoral immune mechanisms (Moisini and Davidson 2009).

### Altered cytokine levels with anti-BLyS and anti-APRIL

The immunization of marmosets with rhMOG/CFA activates both Th1 and Th17 cells, which are both pathogenically relevant in this model, although they seem to operate in different pathways (Kap et al. 2010). We used ELISA to determine the *ex vivo* production of IL-17A, TNF-α and IFN-γ proteins by MNC from blood, spleen and ALN after stimulation with the immunizing rhMOG antigen. Moreover, we determined the levels of mRNA transcripts for several cytokines to assess presence of functional MNC subsets: Th1 (TNF-α and IFN-γ), Th17 (IL-17A), Treg (IL-10) and IL-7 (B cells).

**Fig. 6:**
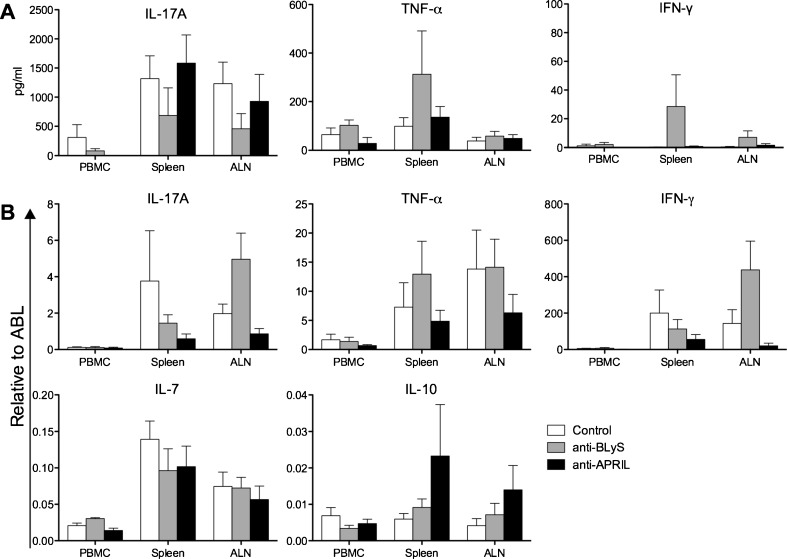
Altered cytokine expression with anti-BLyS and anti-APRIL. **a.** At necropsy MNC were collected from blood and lymphoid organs and cultured for 48 h with a panel of MOG peptides and rhMOG. Levels of IL-17A (left panel), TNF-α (middle panel) and IFN-γ (right panel) were measured in culture supernatants with ELISA (mean ± SEM). Data is only shown of rhMOG stimulated MNC, since no high levels of cytokines were measured in the MOG peptide stimulated cells. **b.** Cytokine mrRNA transcripts of IL-17A, TNF-α, IFN-γ, IL-7 and IL-10 were measured in PBMC, spleen and ALN. Data was normalized to the household gene ABL (mean ± SEM). Mann Whitney *U* test was performed as statistical calculation, but no significant differences were observed

The profiles of cytokines secreted in the rhMOG-stimulated cultures are displayed in Fig. 6a. This graph shows a trend towards reduced IL-17A production in all three analysed compartments of the anti-BLyS-treated animals, compared to both other groups, whereas the levels of TNF-α and IFN-γ were increased. The rhMOG stimulation of MNC from the control and anti-APRIL treated monkeys induced essentially similar secretion levels of IL-17A, TNF-α and IFN-γ. However, due to the inter-individual variation in the outbred model, none of these effects reached statistical significance.

The analysis with cytokine specific qPCR of mRNA directly extracted from blood, spleen and ALN MNC shows inconsistent effects of the anti-BLyS and anti-APRIL treatment (Fig. 6b). Both antibodies induced reduced IL-17A and IFN-γ transcript levels in spleen, whereas levels in ALN are increased in monkeys treated with anti-BLyS mAb but decreased by anti-APRIL treatment. Remarkably, in the anti-BLyS group increased transcript levels for TNF-α were detected in spleen, whereas these were unchanged in ALN. In the anti-APRIL group TNF-α transcripts were reduced in ALN and unaltered in spleen. Interestingly, the transcript levels for IL-10 in spleen and ALN were enhanced in anti-APRIL treated monkeys, whereas the levels were unaltered in spleen and ALN from the anti-BLyS group. Finally, we observed marginally reduced IL-7 mRNA levels in spleen from the anti-BLyS and anti-APRIL treated monkeys, where these cytokines were unaltered in ALN.

In conclusion, both antibodies exerted a modulatory effect on the cytokine profiles of rhMOG-reactive T cells, although the direction of the effect can be opposite for several cytokines.

## Discussion

The rhMOG/CFA induced EAE model in marmosets has presented itself as a useful preclinical model for translational research into MS pathogenesis (’t Hart et al. 2011). On the one hand the model shares well-documented features with the equivalent EAE models in mice and rats. On the other hand the model shares clinical and pathological features with MS, in particular the progressive course and the prominent involvement of grey matter demyelination (’t Hart et al. 2011).

The disease course of the model is not only variable, reflecting the genetic heterogeneity of the marmoset as an outbred species, but also highly complex, affecting the white and grey matter of the brain and spinal cord. As discussed elsewhere (’t Hart et al. 2011), the induction of inflammation and demyelination and the ensuing neurological deficits involves at least two pathways. The “canonical” pathway I seems to be primarily active in the initiation phase of the disease and involves the concerted action of MHC class II-restricted Th1 cells and autoantibodies. *In vivo* activation (Brok et al. 2000)or adoptive transfer (Villoslada et al. 2001) of only the Th1 cells induces small inflammatory lesions without demyelination. Demyelination is effected when monkeys are immunized with B cell epitope containing rhMOG protein or by transfer of monoclonal antibody against MOG protein (Genain et al. 1995). It should be noted that pathway I is not strictly dependent of MOG, as it is also inducible with MOG-deficient myelin, whereas for the activation of pathway II, MOG seems indispensable (Jagessar et al. 2008). The “non-canonical” pathway II becomes likely engaged later in the pathogenic process and involves MHC class I-restricted effector memory natural killer-like cytotoxic T cells (NK-CTL) interacting with Th17 cells (Kap et al. 2008; Jagessar et al. 2010; Jagessar et al. 2012b). The MHC-class II (Caja-DRB*W1201) and MHC class I (*Caja-E*) restriction elements, respectively operating in pathways I (Brok et al. 2000) and II (Jagessar et al. 2012b) are both monomorphic in the common marmoset, explaining why the two pathways can be operational in all animals.

B cells have a pathogenic contribution to both pathways. The main role of B cells in the canonical pathway I is the production of autoantibodies against myelin, which elicit cellular and/or complement dependent cytotoxicity reactions. The primary role in the non-canonical pathway II, which is independent of autoantibodies, is antigen presentation to the NK-CTL that induces demyelination of grey and white matter without detectable involvement of autoantibodies. The latter role of B cells was shown in two independent experiments. One set of experiments demonstrated that depletion of B cells with the anti-CD20 antibody HuMab7D8 from marmosets at 21 days after the immunization with rhMOG/CFA abrogates the development of EAE symptoms (Kap et al. 2010) as well as of EAE pathology in the grey and white matter of the brain and spinal cord (Kap et al. 2011). This highly robust effect was associated with impaired activation of the cellular autoimmune mechanisms that mediate disease progression. A second set of experiments was performed in the novel MOG34-56/IFA EAE model in which white and grey matter demyelination is induced by peptide-specific NK-CTL without a detectable influence of autoantibodies (Jagessar et al. 2010; Jagessar et al. 2012b). In the same MOG34-56/IFA EAE model it was observed that depletion of CD20+ B cells from 21 days after the immunization abrogates the development of EAE symptoms and pathology (submitted for publication).

We report here the effect on the rhMOG/CFA EAE model of two monoclonal antibodies against essential factors for B cell survival and differentiation. BLyS and APRIL have partially overlapping roles in B cell survival and differentiation involving at least 3 receptors, i.e. BCMA, TACI and BR3, which recognize homo-trimeric forms of BLyS and APRIL (Miller et al. 2006). BLyS binds to all three receptors, but with highest affinity for BR3. APRIL does not bind BR3 and has about 100-fold higher affinity for BCMA than BLyS. TACI binds both factors with comparable affinity.

The results of the current study show that anti-BLyS and anti-APRIL administration to rhMOG/CFA immunized marmosets induced depletion of CD20+ B cells from blood at a similar rate and depth. Similar to observations in cynomolgus monkeys the depletion with anti-BLyS mAb was less fast or profound than with anti-CD20 mAb (Halpern et al. 2006). In the monkeys treated with anti-BLyS, but not in those treated with anti-APRIL we observed reduction of CD20+ B cells in spleen and lymph nodes. With qPCR analysis it was also shown that CD19 transcript levels were reduced in spleen and ALN of anti-BLyS as well as anti-APRIL treated monkeys. It is difficult to explain these discrepancies although it should be noted that CD20 is an exclusive marker of the B cell lineage, whereas CD19 is also expressed by the follicular dendritic cells that populate lymphoid organs. Interestingly, we observed no depletion by anti-BLyS or anti-APRIL mAb of the CD20 + CD40+ subset. This subset is actively engaged in the EAE pathogenesis as illustrated by the finding that administration of antagonist anti-CD40 mAb suppressed EAE development in the rhMOG/CFA model (Boon et al. 2001). However, disease activity within the CNS was only partly suppressed. This is a remarkable difference with the robust clinical (Kap et al. 2010) and pathological (Kap et al. 2011) effect of anti-CD20 mAb treatment in the rhMOG/CFA model, in which complete depletion of the CD20 + CD40- and CD20 + CD40+ subsets was observed. Collectively, these data show that for an optimal clinical effect of B cell targeting antibodies in the rhMOG/CFA model probably both subsets of B cells have to be depleted.

The separate antibody-mediated inhibition of BLyS and APRIL exerted comparable clinical effects in the EAE model, although several differences at the immunological and pathological level were observed. Neutralization of BLyS or APRIL caused a significantly delayed onset of overt clinical EAE (EAE score 2.0, ataxia). However, the effect on the time to the study end-point (EAE score 3.0, hemi-/paraplegia) was statistically significant only for the group treated with anti-BLyS antibody. Moreover, in the monkeys treated with anti-BLyS, but not in those treated with anti-APRIL, we observed significantly reduced demyelination of spinal cord. These discrepant effect may be explained by the fact that the anti-BLyS antibody targets not only processes in the periphery, i.e. B cell survival, but also in the CNS, i.e. neuronal outgrowth. BLyS is produced within the CNS by reactive astrocytes and exerts via NgR1 similar effects on neurons as the natural ligand Nogo66 (Zhang et al. 2009). To our knowledge, such non-immunological effects in the EAE model have not been reported for APRIL.

The two mAbs were found to modulate, albeit in a different manner, cellular autoimmune reactions involved in the EAE pathogenesis. This was observed in regular proliferation assays using MNC isolated from different immune compartments; response patterns of T cell subsets assessed by CFSE vital dye dilution and the cytokine profiles. We observed in the anti-BLyS-treated group enhanced proliferation of MNC from blood and lymphoid organs against MOG peptides 14–36 and 24–46, which both contain the epitope MOG24-36 that is presented by a ubiquitously expressed monomorphic Caja-DRB*W1201 allele to Th1 cells (Brok et al. 2000; Doxiadis et al. 2006). The higher production of TNF-α and IFN-γ by the rhMOG-stimulated splenocytes supports this conclusion. We observed lower production of IL-17A than in the other two groups i.e. anti-APRIL and the controls. Phenotyping of proliferating cells via CFSE vital dye dilution and monoclonal antibody staining shows that CD3+ T cells from anti-BLyS treated monkeys displayed higher proliferative activity than those from the other two groups, which are localized mostly within the CD4 + CD56+ subset. However, the rhMOG and MOG34-56-stimulated proliferation are localized mainly in the CD8+, CD4 + CD8+, CD8 + CD56+ subsets. Importantly, the reactivity of the CD4 + CD8 + CD56+ subset to antigenic stimulation, which is the main source of the autoreactive T cells cytotoxic activity in the marmoset EAE model (Jagessar et al. 2010; Jagessar et al. 2012b), is not increased by the treatment.

In the monkeys treated with the anti-APRIL mAb we did not observe marked differences with the control monkeys at the level of proliferation by MNC from blood, spleen or lymph nodes, neither in the TCPA test nor with CFSE vital dye dilution. However, the cytokine analyses showed the lowest expression of pro-inflammatory cytokines IL-17A, IFN-γ and TNF-α mRNA transcripts concomitant increased expression of anti-inflammatory IL-10 mRNA in this group.

In conclusion, the treatment with anti-BLyS and anti-APRIL antibody, which was started at a relatively late stage (day 21 post immunization), results in a significant delay of EAE onset and progression. Due to the heterogeneity between individual monkeys, which is inherent to this outbred model, differences between investigated variables in the three groups often did not reach statistical significance. This implies that they should be interpreted with caution as only trends can be indicated. The clinical effect of the therapeutic antibodies seems to be based on different mechanisms. The anti-BLyS treated group displays reduced antibody levels against rhMOG and MOG peptides, together with reduced Th17 and increased Th1 activation. We hypothesize that treatment with the anti-BLyS antibody may impair both the canonical and the non-canonical pathogenic pathway. Besides this immunomodulatory effect, the depletion of BLyS could also have consequences inside the CNS, such as removal of axonal outgrowth inhibition. For the anti-APRIL antibody such central effects are not known, which may explain why the disease progression after initiation (score 2.0 to 3.0) is not significantly affected. However, the antibody has modulatory effects on the autoimmune reaction, most notably a reduction of Th1 and Th17 signature cytokine transcripts and an increase of IL-10 mRNA. This may suggest that the inhibitory activity of the anti-APRIL mAb mainly affects pathway I (B cells/auto-antibodies), and is based on skewing from a pro- to anti-inflammatory T cell profile. Further research should demonstrate whether this is indeed the case.
